# Alzheimer’s and other dementias in Canada, 2011 to 2031: a microsimulation Population Health Modeling (POHEM) study of projected prevalence, health burden, health services, and caregiving use

**DOI:** 10.1186/s12963-016-0107-z

**Published:** 2016-11-03

**Authors:** Douglas G. Manuel, Rochelle Garner, Philippe Finès, Christina Bancej, William Flanagan, Karen Tu, Kim Reimer, Larry W. Chambers, Julie Bernier

**Affiliations:** 1 Health Analysis Division, Statistics Canada, Ottawa, Ontario Canada; 2 Ottawa Hospital Research Institute, Ottawa, Ontario Canada; 3 Department of Family Medicine, University of Ottawa, Ottawa, Ontario Canada; 4 Bruyère Research Institute, Ottawa, Ontario Canada; 5 School of Public and Population Health, University of Ottawa, Ottawa, Ontario Canada; 6 Institute for Clinical Evaluative Sciences, Ottawa and Toronto, Ottawa, Ontario Canada; 7 Public Health Agencies of Canada, Ottawa, Ontario Canada; 8 Department of Family Medicine, University of Toronto, Toronto, Ontario Canada; 9 BC Ministry of Health, Victoria, British Columbia Canada; 10 Alzheimer’s Society of Canada, Toronto, Ontario Canada; 11 Faculty of Health, York University, Toronto, Ontario Canada; 12 Department of Clinical Epidemiology and Biostatistics, McMaster University, Hamilton, Ontario Canada

**Keywords:** Dementia, Alzheimer disease, Health planning, Computer simulation, Population dynamics, Home care services, Quality of life, Outcome assessment (Health Care), Health status, Time factors

## Abstract

**Background:**

Worldwide, there is concern that increases in the prevalence of dementia will result in large demands for caregivers and supportive services that will be challenging to address. Previous dementia projections have either been simple extrapolations of prevalence or macrosimulations based on dementia incidence.

**Methods:**

A population-based microsimulation model of Alzheimer’s and related dementias (POHEM:Neurological) was created using Canadian demographic data, estimates of dementia incidence, health status (health-related quality of life and mortality risk), health care costs and informal caregiving use. Dementia prevalence and 12 other measures were projected to 2031.

**Results:**

Between 2011 and 2031, there was a projected two-fold increase in the number of people living with dementia in Canada (1.6-fold increase in prevalence rate). By 2031, the projected informal (unpaid) caregiving for dementia in Canada was two billion hours per year, or 100 h per year per Canadian of working age.

**Conclusions:**

The projected increase in dementia prevalence was largely related to the expected increase in older Canadians, with projections sensitive to changes in the age of dementia onset.

**Electronic supplementary material:**

The online version of this article (doi:10.1186/s12963-016-0107-z) contains supplementary material, which is available to authorized users.

## Research in context

### Systematic review

Two main approaches have been used to project the burden of Alzheimer’s and related dementia: extrapolation of current prevalence, and macrosimulation of disease incidence and mortality. Microsimulation modeling has been suggested as an approach to improve dementia projections.

### Interpretation

We present the first, to our knowledge, population model of Alzheimer’s and related dementia for projecting incidence, prevalence, and related outcomes: the Population Model for Neurological Diseases (POHEM:Neurological). POHEM:Neurological projected a dementia burden that was modestly higher than most previous projections. The microsimulation approach used in this study offers three benefits: comparisons of people with dementia to the general population (e.g., the ability to examine how caregiving for people with dementia will influence total caregiving); greater specificity (e.g., the ability to incorporate a range of factors that influence dementia prevalence); coherent application of a range of data (e.g., the same dementia definition and measure of disease severity to generate a wide range of dementia outcomes).

### Future directions

POHEM:Neurological could be adapted for use in other jurisdictions. The addition of dementia risk factors could improve dementia incidence projections. The addition of these risk factors is feasible, given many risk factors are the same as those for cardiovascular disease and are included in the POHEM:Cardiovascular disease model.

## Background

The prevalence of Alzheimer’s and other dementias is projected to increase considerably in all countries as a reflection of the world’s aging population [1–4]. The World Health Organization estimates the global prevalence of dementia to be 47.5 million, and this number is expected to increase substantially in the years ahead, reaching 135 million by 2050 [4, 5].

The socioeconomic needs that dementia exerts on society as a whole has made it a public health priority [6]. Furthermore, there are concerns regarding the potential increase in the magnitude of dementia prevalence and the level of care required for each person with dementia [3, 6]. Population-based dementia models have been created to support planning by projecting the number of people living with dementia and by allowing the examination of counterfactual scenarios that may ameliorate or exacerbate dementia’s societal burden.

However, current dementia models have been criticized as being overly simplistic representations of dementia progression. Ideally, population models should start from people who do not have disease and project incident cases, then move onward to describe disease progression from mild to more severe disease or to death [3]. However, previous dementia projections have either been extrapolations of current prevalence trends, which lack incidence rates altogether, or macrosimulation (cell-based) studies, which use a constant incidence and mortality rate, among other simplifying assumptions.

Norton et al. have called for the development of population-based microsimulation models to address limitations from previous projection models [3]. These authors specifically cite Canada’s Population Health Model (POHEM) – developed for chronic diseases such as diabetes, cardiovascular disease, and arthritis – as an example of a microsimulation model that more robustly includes population dynamics (birth, deaths, and migration) and disease progression (from disease-free to disease with varying levels of health-related quality of life or health care use) [7–9]. As well, more complex modeling approaches have the benefit of synthesizing a wide range of data sources to generate a more comprehensive viewpoint of the disease, meaning that microsimulation models provide insight for complex connected systems [10].

We sought to project the prevalence of dementia and the related health and health care burden in Canada from 2011 to 2031 by adapting Canada’s current Population Health Model (POHEM) to create a new POHEM:Neurological model and supporting software. Using Statistics Canada’s POHEM framework, we created a population-based longitudinal microsimulation model named POHEM:Neurological. The model was created as part of Canada’s National Population Health Study of Neurological Conditions (NPHSNC), which included 13 supporting research projects and three national surveys that examined 13 different neurological conditions [11]. The current project had two goals: i) to project dementia burden in Canada from a societal perspective that included the health impacts as well as direct and indirect heath care (out-of-pocket costs and informal caregiving), and ii) to synthesize the wide range of dementia information from projects within the NPHSNC.

This study had advisory input from people living with neurologic conditions, their caregivers, clinicians, and individuals with policy expertise [11]. The project’s advisors and research team created the model specification, which included the model’s purpose, its overall structure, and data sources. The requested model attributes included the following:

- *Population-based* – reflecting the Canadian population including important sub-populations based on factors such as age, sex, and provincial regions.
- *Open population* – allowing the population to change over time to reflect historical and projected births, deaths, immigration, and emigration.
- *Consistent and coherent* – using a consistent definition of dementia and health-related quality of life (HRQOL) throughout the model. All outcome measures – from the onset of dementia to death – are coherently linked to the definitions of dementia and HRQOL.
- *Predictive accuracy* – able to generate accurate (or well-calibrated) projections.
- *Useful for population health planning* – can be used to estimate future dementia burden including direct and indirect health care costs and caregiver burden.
- *Flexible and robust* – provision to further develop the model. Risk factors for the development of dementia were explicitly excluded from the current study. However, there is provision for the inclusion of risk factors in future dementia modeling [7].

## Methods

### Population Health Model (POHEM) framework

Briefly, POHEM is an empirically grounded, longitudinal microsimulation model of diseases and risk factors representing the lifecycle dynamics of the Canadian population [12, 13]. The basic unit of analysis is individual people, or “actors,” whose life course is modeled in continuous time using a Monte Carlo approach. The dynamic simulation recreates the Canadian population at a given point in time (both historically and in the future) and ages it, one actor at a time, until each actor’s death.

### Model development

**Fig. 1:**
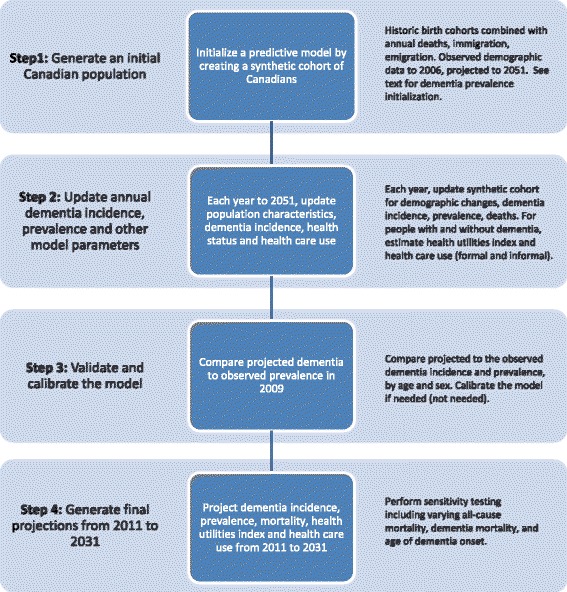
Process for Population Health Model (POHEM):Neurological

Figure 1 shows the four steps that make up the process of microsimulation model development: initialization, yearly updates, model validation, and projection. Canadian population-based data sources were used throughout the model (see Additional file 1).

### Initialization

POHEM:Neurological was initialized using the same approach that was used for POHEM cancer modeling (called OncoSim) [14]. The purpose of model initialization was to create model “actors” to reflect the Canadian population, both historic observations and further population growth projections [15].

Initialization began with historic birth cohorts from 1872, which were subject to observed historic death rates. Migration (immigration and emigration) was added to the birth cohorts, also reflecting the historic observed or estimated events. The birth cohorts used observed data up to 2006, with projected births, deaths, and migration that followed standard Canadian population projections (mid-growth scenario), as estimated by Statistics Canada [15]. High- and low-growth scenarios were used in sensitivity testing (described later).

#### Yearly updates of actors’ health profiles

An actor’s health profile consists of six main characteristics: (i) demographics (e.g., age, province of residence); (ii) dementia status; (iii) health status; (iv) presence of an informal caregiver; (v) health care costs; and (vi) mortality (date of death). Each actor’s health profile was updated throughout the year, either at the occurrence of an event (e.g., birthday, date of diagnosis of dementia) or at the change of the calendar year, depending on the profile characteristic. All health profile characteristics were calculated and modeled for people with and without dementia (see Additional file 1 for data sources).

#### Dementia status: incidence, prevalence and mortality

Two steps were followed to generate prevalent dementia actors. First, sex- and age-specific dementia incidence rates were applied to the model’s synthetic Canadian population for each year, both historical and projected. Dementia incidence rates were estimated using a case definition algorithm with a sensitivity of 79.3 % and specificity of 99.1 % among individuals age 65 years and older (see Additional file 1 for ascertainment diagnostic codes, algorithm, and incidence rates), [16, 17] and applied to administrative health data from the province of British Columbia. Actors where classified as being diagnosed with dementia based on each actor’s risk of developing dementia at the beginning of each calendar year. Incident dementia cases accumulated over time to generate prevalent cases of dementia.

Second, dementia-specific mortality risk was applied to actors with dementia. The dementia mortality risk was a product of a mortality ratio for people with dementia multiplied by the baseline mortality rate for the Canadian population within POHEM. The general population’s mortality rate gradually decreased over time, reflecting the projected mortality (life expectancy) using birth cohorts and the Lee-Carter model as estimated by Statistics Canada [18]. This means that the projected mortality for people with dementia decreased at the same rate as for Canadians living without dementia. The mortality hazard ratio for people with dementia was estimated using the same case definition and data used to estimate dementia incidence.

#### Health status

**Fig. 2:**
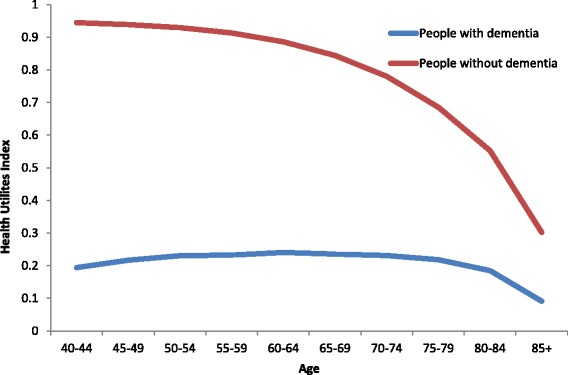
Health status (Health Utilities Index Mark 3) for people with and without dementia, Canada, 2011

The Health Utilities Index Mark 3 (HUI3) is a utility-based measure that reflects health states ranging from perfect health (HUI3 = 1.0) through death (HUI3 = 0), including states considered to be worse than death (minimum HUI3 = -0.36), thereby allowing a wide range of severity levels and applications [19]. The HUI3 was used because: i) there is a consistent use of the HUI3 across many Canadian dementia data sources; and ii) the HUI3 is routinely measured for Canadians without dementia, including the general population and people with other chronic disease, thereby allowing an assessment of the incremental burden of dementia and/or counterfactual scenarios. See Fig. 2 for age-specific mean HUI3 for people with and without dementia.

#### Informal caregivers

At the end of every calendar year, the likelihood that an actor would receive informal caregiving for the following year was based on the actor’s age, dementia status, and health status (HUI3). If an actor was assigned the presence of an informal caregiver, additional characteristics were also assigned: (i) hours of care received; (ii) health status of their caregiver; and (iii) out-of-pocket expenses incurred by caregivers.

#### Health care costs

The health care costs included formal health care costs, out-of-pocket costs, and informal care by caregivers (hours of care per week). Formal health care costs were examined for seven health sectors: (i) hospitalization; (ii) physician services; (iii) prescription drugs; (iv) rehabilitation hospitals; (v) formal (paid) home care; (vi) residence and care in a long-term care facility; and (vii) assistive devices. Formal health costs were based on whether an actor was an incident case (within the first 12 months following incidence) or prevalent case (one year or more since incidence) of dementia. Out-of-pocket expenses were those not covered by private insurance or provincial health care plans, such as the cost of prescription and over-the-counter medications, assistive devices, rehabilitation therapy such as physical or occupational therapy, home care services. See Additional file 1 for details.

#### Validation

Model validation focused on calibration and was performed by comparing projected to observed estimates for a given calendar year [20, 21]. We compared the projected estimates of dementia prevalence and deaths to the rates observed in British Columbia in 2009, the same province in which the age and sex-specific dementia incident rates were estimated. Validating model-predicted estimates against an external reference population was the preferred approach of assessing predictive accuracy of the baseline model [20, 21]. While the British Columbian population did not meet the criteria of an external population, the generated predictions were estimated using a series of historic birth cohorts, which was a considerably different approach than what was used to estimate dementia prevalence using administrative data. The model initialization showed uncalibrated estimated dementia prevalence that closely approximated the observed age- and sex-specific prevalence in 2009 (see Additional file 1); therefore calibration was unnecessary.

#### Projection

Model parameters were projected through 2031 and beyond, including: (i) incident and prevalent dementia cases in Canada; (ii) years of life lost (YLL) with dementia; (iii) health-adjusted life years lost (HYLL); (iv) direct health care costs – including costs for each of the seven sectors; (v) out-of-pocket expenses; and (vi) hours of informal caregiving. In addition, three parameters were projected from the perspective of caregivers for dementia: (i) hours of caregiving; (ii) out-of-pocket expenses; and (iii) health status (HUI3).

YLL was defined as the difference between the age of death of an actor with dementia compared to the age of death if that actor did not develop dementia. To obtain the latter, we set the relative mortality hazard for dementia to 1.0 and recalculated age of death: YLL was the difference in age of death between the two calculations. HYLL was calculated in a similar manner, with health-adjusted life years estimated from the product of years of life lived (i.e., age of death) multiplied by the annual HUI3 for each actor over their lifetime.

Sensitivity analyses were performed to examine the influence of different model parameters on the projected burden of dementia. These included: (i) high and low projected Canadian life expectancy, as per Statistics Canada population projections; [15] (ii) delayed dementia onset (lower dementia incidence); and (iii) lower risk of dementia death.

#### Microsimulation model and statistical analyses

The POHEM model was generated using MODGEN (Model Generator), a microsimulation programming language developed and supported by Statistics Canada [22].

## Results

### Projected dementia prevalence

At baseline in 2011, there were 340,000 people living with dementia in Canada, a number which was projected to approximately double to 674,000 people by 2031 (1.98-fold increase) (Table 1). There was a smaller increase in the prevalence rate for older people (e.g., age 80 years and older) compared to younger people (1.06-fold increase by 2031 versus 1.55-fold increase), a reflection of the overall aging of the Canadian population.

**Table 1:** Dementia prevalence (cases per 1000 people), Canada, 2011 to 2031, by sex

	2011	Baseline		Sensitivity analyses, ratio (2031:2011)		
		2031	Ratio (2031:2011)	Higher Canadian life expectancy^a^	Decreased dementia mortality^b^	Delayed incidence^c^
Number of people with dementia						
Total	340,000	674,000	1.98	2.08	2.09	1.10
Males	142,000	291,000	2.05	2.16	2.15	1.13
Females	198,000	383,000	1.93	2.02	2.04	1.07
Prevalence rate (per 1000 people)						
Age 40 years and older						
Total	20.0	31.0	1.55	1.61	1.63	0.85
Males	17.3	27.6	1.60	1.67	1.68	0.88
Females	22.5	34.1	1.52	1.57	1.59	0.83
Age 80 years and older						
Total	177	187	1.06	1.08	1.12	0.62
Males	171	182	1.06	1.09	1.12	0.62
Females	180	191	1.06	1.08	1.12	0.61

^a^Higher life expectancy based on projections by Statistics Canada

^b^10% lower mortality hazard among those with dementia, relative to baseline relative mortality hazard

^c^Delay age-specific incidence of dementia by 5 years, relative to baseline incidence rates

When evaluating the burden of dementia, the most sensitive model parameter for dementia prevalence projection was dementia incidence. When dementia incidence was delayed by 5 years, there was only a 1.10-fold increase in dementia cases (374,000 people projected to be living with dementia in 2031) as opposed to the 1.98-fold increase projected by the base model. Changes to the projected Canadian life expectancy (affecting those with and without dementia) had a modest impact on dementia projections, with only a small increase in the projected number of people living with dementia in 2031 under a high life expectancy scenario (2.08-fold increase in people living with dementia). Improved (decreased) relative mortality for people living with dementia had a similar modest increase in dementia projections, with a 10 % decrease in the dementia-specific mortality rate resulting in a 2.09-fold increase in dementia prevalence by 2031.

### Projected health burden

Table 2 shows the projected health burden of dementia. The projected number of deaths among people with dementia increased from 2011 to 2031 at a somewhat lower rate than the increase in dementia prevalence (1.70-fold increase in the number of dementia deaths compared to 1.98-fold increase in the number of people living with dementia). The relatively smaller increase in the number of deaths was a consequence of a projected decrease in mortality for the general population, which itself resulted in a decrease in the death rate among people with dementia (146 deaths/1000 people in 2011 versus 125 deaths/1000 people in 2031). For persons with dementia who died in 2011, there were 3.4 YLL and 3.3 HYLL: extrapolated to the population level, 1.2 million YLLs and 1.1 million HYLL were lost due to dementia. There was a modest projected reduction of YLL and HYLL by 2031.

**Table 2:** Projected burden and informal caregiving for people living with dementia, Canada, 2011 to 2031

	2011	Baseline scenario, 2031		Decreased dementia mortality^a^, 2031		Delayed incidence^b^, 2031	
		Estimate	Ratio	Estimate	Ratio	Estimate	Ratio
Number of people with dementia	340,000	674,000	1.98	709,000	2.09	374,000	1.10
Deaths	50,000	85,000	1.70	83,000	1.66	52,000	1.04
Mortality rate (deaths/1000)	146	126	0.86	118	0.81	138	0.95
Years of life lost	3.4	3.2	0.96	2.9	0.86	2.9	0.87
Health-adjusted years of life lost	3.3	3.2	0.98	3.2	0.97	2.9	0.88
Informal care							
People receiving care	261,000	522,000	2.00	552,000	2.11	291,000	1.11
Hours per year (millions)	1,000	2,000	2,100	2,100	1.13	1,130	1.13
Hours per year (per person age 25 to 65 years)	52.3	100.4	106.5	106.5	1.08	56.6	1.08
Receiving no care (%)	23.2	22.5	0.97	22.2	0.96	22.0	0.95
Less than 7 h/week (%)	<0.1	<0.1	1.34	<0.1	1.28	<0.1	1.03
7 to 14 h/week (%)	0.1	0.1	1.02	0.1	0.97	<0.1	0.77
15 to 70 h/week (%)	26.5	26.3	0.99	26.0	0.98	24.3	0.92
71 or more hours/week (%)	50.3	51.1	1.02	51.7	1.03	53.6	1.07

^a^10% lower mortality hazard among those with dementia, relative to baseline relative mortality hazard

^b^Delay age-specific incidence of dementia by 5 years, relative to baseline incidence rates

For people living with dementia in 2011, 77 % were estimated to be receiving informal care (261,000 people), with less than a 1 % projected increase in the proportion receiving care by 2031. The small change was a reflection of model assumptions, namely that the factors affecting the receipt of care (i.e., HUI3 values) did not change over time in our model. However, the total number of people with dementia receiving care was projected to increase 2-fold to 522,000 in 2031, reflecting the increase in the number of people living with dementia. There was a corresponding increase in the number of caregiving hours; in 2031, a projected 2.0 billion informal caregiving hours were utilized by persons with dementia. The caregiving hours per working-age person (ages 25 to 65 years) were projected to increase from 52.3 to 100.4 h per week.

### Projected health care use

Table 3 shows that the direct health care cost for people with dementia was $9.2 billion CDN in 2011 ($27,000 per-person cost), which was projected to increase to $18.2 billion in 2031. In sensitivity analysis, the 2031 costs varied from $10.3 billion with a delay in dementia onset to $19.2 billion with a reduction in dementia mortality hazard. The highest cost sectors were long-term care (46 % of total cost) and hospitalization (27 % of total cost).

**Table 3:** Health care costs (millions of dollars) among people with dementia, Canada, 2011 to 2031, by sector

Health care sector	2011	2031		
		Baseline	Decreased dementia mortality^a^	Delayed incidence^b^
Physician services	728	1,440	1,510	798
Hospitalization	2,560	5,000	5,210	2,790
Drug	1,000	2,000	2,100	1,090
Rehab	85	169	180	96
Assistive devices	80	160	170	90
Home care	580	1,160	1,220	646
Long-term care	4,160	8,300	8,830	4,740
Total cost	9,200	18,210	19,210	10,250

^a^10% lower mortality hazard among those with dementia, relative to baseline relative mortality hazard

^b^Delay age-specific incidence of dementia by 5 years, relative to baseline incidence rates

## Discussion

We created a population-based microsimulation model of dementia in Canada and projected 13 health and health care outcomes for people living with and without dementia. Using a single unified model, it is possible to estimate and project most measures in typical national reports such as the *United States Alzheimer’s disease facts and figures* [23].

We found that, between 2011 and 2031, the burden of dementia for most outcomes was projected to approximately double. By 2031, the number of hours of informal care per person (per Canadian of working age, 25-65 years) was projected to be 100 h per year, or approximately 2.7 h per workweek (at 37.5 h per week).

### Current study in perspective

To our knowledge, this is the first population-based microsimulation projection of dementia. That stated, microsimulation methods have been used to estimate current population and individual lifetime cost [24, 25]. As in most other studies, we found that the burden of dementia was projected to increase substantially as a consequence of an “aging” population, meaning a population with a higher proportion of older people compared to younger people [2, 3, 26]. However, our study’s projections showed a more rapid increase compared to projected increases in other developed countries, where dementia is projected to increase by 40 % in Europe and 63 % in North America within a 20-year period [26].

Without a detailed comparison of studies, it is difficult to identify why we project a more rapid increase in dementia. Potential differences in models may relate to underlying demographic changes, dementia-specific mortality, or differences in dementia incidence. Difference in the demographic projections likely contributed to important differences in the projected increase in dementia in our study compared to projections in the United States and Europe. For example, no other dementia model, to our knowledge, has considered how all-cause or dementia mortality will change over time. The expected decrease in both all-cause and dementia mortality will increase survival time for people living with dementia and thus increase projected prevalence. Mortality projections for dementia in our study started with mortality projections for the general Canadian population that used a well-established method used by national statistical agencies [15, 18]. The relative mortality hazard of dementia was calculated using the same dementia ascertainment approach that was used to estimate dementia incidence.

In our study, dementia incidence (or age of dementia onset) was shown to have a strong influence in the projected increase in the rate of dementia. However, the approach and assumptions of assessing dementia incidence should not influence the rate of dementia increase. We used a definition of dementia that relied on physician-coded diagnoses in health administrative data, whereas many other studies and most reviews of dementia prevalence used a broader definition of dementia that included undiagnosed dementia cases [26]. Differing ascertainment approaches will affect the *absolute level* of dementia prevalence but not the *rate of increase* of dementia, unless there is a change in the proportion of cases that are diagnosed. Approximately 30 % of dementia cases in Canada are not diagnosed, and the identification approach using administrative (with imperfect sensitivity) will further under-ascertain dementia by 10 % [27]. The definition of diagnosed dementia in our study allowed for a coherent assessment of burden for all dementia outcomes including mortality, health status (disease severity), and health care use (both formal and informal care), and reflected the preferences of the study’s advisory panel. Furthermore, the ascertainment approach is widely used in Canada for chronic disease surveillance, including within the larger (Canadian) National Population Study of Neurological Conditions, as well as the Public Health Agency of Canada’s Canadian Chronic Disease Surveillance System. A lack of dementia diagnosis coherence in modeling studies can result is poor calibration when, for example, one definition is used for disease incidence but mortality risk is estimated using a different definition.

**Fig. 3:**
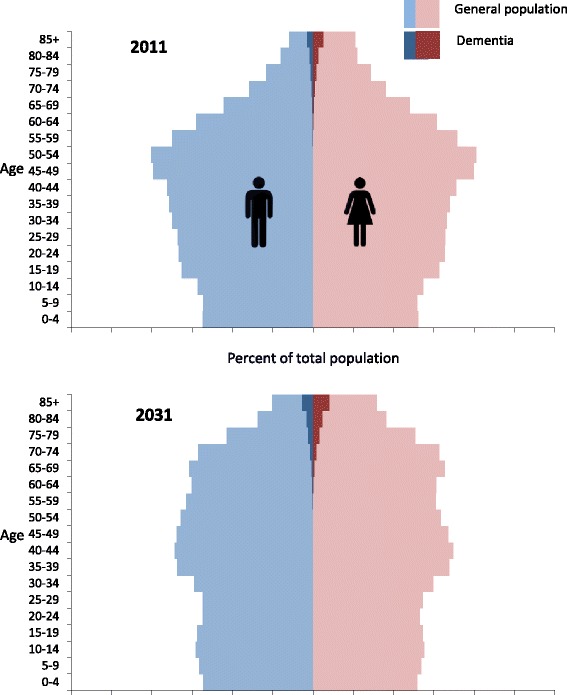
Canadian population and proportion of people with dementia

Lastly, dementia prevalence varies considerably between studies depending on whether the rate is reported for all ages or for specific age cutoffs. Care is required when comparing projected prevalence between studies to ensure the same age groups are considered. Studies that report dementia for only older ages will generally report higher rates of dementia increase compared to studies that project total dementia. We present the full age pyramid in Fig. 3 to provide insight into the projected Canadian population structure and how it will influence the number of people with dementia.

The microsimulation population approach has several additional advantages over previous modeling approaches. First, within POHEM:Neurological there are the same health and health care outcomes for people with and without dementia, including people with other chronic conditions. This population perspective builds on our existing understanding of health for the entire population and allows for a comparison with people without dementia or other chronic diseases. Second, a microsimulation approach allows for greater breadth and complexity compared to other modeling strategies. We included a wide range of health outcomes, which allows for a more comprehensive understanding of the burden of dementia. The greater complexity also allows for a more realistic representation of dementia burden. For example, we examined caregiving use and out-of-pocket expenses that varied by age, sex, and health status (HUI3).

As previously stated, the data consistency of the dementia model was a strength. In addition to a consistent case definition of dementia, we also used a consistent health status or disease severity measure, the HUI3, across different data sources for both people with dementia and their caregivers.

POHEM:Neurological also has limitations. As models become increasingly complex, they become difficult for users to fully understand, leading to a common concern that they seem like “black boxes” which are difficult to assess [10]. These concerns are well-founded. However, POHEM facilitates transparency using several approaches. First, the predictive accuracy of model estimates can be validated. Validation is usually performed with a historic “wash in” period that compares model-predicted estimates to observed estimates. We found that model-projected prevalence using historic data closely approximated observed prevalence. Second, the assumptions in POHEM:Neurological can be assessed. Many dementia projections are extrapolations of historic prevalence trends [3]. Extrapolations are simple and intuitive – and thereby *appear* to be easy to understand. However, extrapolation combines so many factors that it can be difficult to understand what influences their projected estimates. By comparison, POHEM:Neurological generates prevalence estimates combining population growth parameters (migration, births, deaths), dementia incidence, and dementia deaths. Each parameter can be examined and/or modified separately.

Currently, dementia projection models, including POHEM:Neurological, do not consider preventable risk factors. Norton et al. recommended that predictive microsimulation models be used for population-based dementia projections, in part because microsimulation models are well-suited for inclusion of dementia risk factors. Norton et al. singled out the POHEM family of microsimulation models because the modeling framework already includes projections of most well-established preventable risk factors for dementia [3]. For example, Barnes and Yaffe estimated that 50 % of Alzheimer’s disease is attributable to diabetes, hypertension, obesity, depression, physical activity, smoking, and low education [28]. Currently, the cardiovascular disease module of POHEM includes and projects all of these risk factors except depression. However, we felt that more development studies are required to understand the predictive nature of these risk factors on dementia incidence.

Lastly, a limitation of the study is the lack of confidence or uncertainty intervals of model estimates. In lieu of uncertainty estimates, we performed sensitivity analyses of the main model parameters and found that dementia incidence and age of diagnosis had a strong influence on prevalence. As well, we present the distribution of informal caregiving hours received (Table 2) to demonstrate how model projections using a microsimulation approach can readily include distributions of model inputs and/or outputs. The approach to estimate uncertainty in models has not been well established, but there is recognition that it should include concepts of statistical error, uncertainty from model specification and approach, and the distribution of model parameters [21]. In the era of models generated from large data, such as POHEM:Neurological, most important uncertainty likely originates from model specification and distribution of model parameters.

## Conclusions

To our knowledge, this study is the first to project dementia using a microsimulation model. Microsimulation models of dementia have the potential to produce improved estimates for planning. We projected a more rapid increase in dementia prevalence by including projections of underlying all-cause mortality rates. The microsimulation framework more readily allows for multiple dementia outcomes. For example, this study is the first, to our knowledge, to project the need for caregiving, based on projections of dementia prevalence and disease severity. In addition, we project a wide range of outcomes not previously reported, including health care costs by sector and health-adjusted life years lost. Microsimulation models are also well-suited for scenario testing. It appears that prevention could play an important role in reducing dementia burden, given the strong influence of delaying disease incidence.

The POHEM:Neurological model shows that it is feasible to develop a well-calibrated population-based microsimulation model of dementia that provides a robust framework for further development and application for planning. POHEM:Neurological could be used by other jurisdictions by replacing model parameters such as Canadian dementia incidence.

## Additional file

Additional file 1: Details of data sources. (PDF 268 kb)
