# Supplementary material for: Alzheimer’s and other dementias in Canada, 2011 to 2031: a microsimulation Population Health Modeling (POHEM) study of projected prevalence, health burden, health services, and caregiving use
Source: Popul Health Metr. 2016 Nov 3;14:37. doi: 10.1186/s12963-016-0107-z (PMC5095994; doi:10.1186/s12963-016-0107-z)
Supplement: Additional file 1: — Details of data sources. (PDF 268 kb) [file 12963_2016_107_MOESM1_ESM.pdf]

Additional file 1

Data sources

Source of date used to ascertain dementia incidence, prevalence, mortality, health status, health care use and caregiving.

Exhibit 1 - Data sources

Model parameter	Canadian source
**Demographics**<br>(historic and projected births, deaths, migration)	Statistics Canada
**Dementia incidence and mortality rate**	Administrative data (British Columbia)
**Health Status**<br>(Health Utilities Index Mark III)	For people without dementia: National Population Health Survey
	For people with dementia: Survey on Living with Neurologic Conditions in Canada (2011 SLNCC)
**Caregiving**	General Social Survey (2012 GSS)
**Health care use**	Health administrative data (Ontario and British Columbia)

Ascertainment of dementia

Exhibit 2 - International Classification of Disease (ICD) coding

Condition	ICD system	ICD codes
**Alzheimer's/ Dementia**	10	G30, F00, F01, F02
	9	046.1, 290, 294, 331.0, 331.1, 331.5
	9CM	046.1, 290, 294, 331.0, 331.1, 331.5

Incident rates were estimated for people age 40 and older using population-based health administrative data for the province of British Columbia from 1992/93 to 2009/10. The identification algorithm was 3 physician visits, 30 days or more between visits in a 2-year period, or 1 hospitization, or 1 perscription for people over age 65 years. The algorithm sensitivity and specificity was 79.3% and 99.8% for people age 65 years and older and 75.0% and 99.8% for people age for people 20 years and older.[1] Physician visits were ascertained using the Medical Services Plan database includes up to three International Classification of Disease, version 9 (ICD-9) codes from each physician visit. Hospitalization data housed in the Canadian Institute for Health Information Discharge Abstract Database (CIHI DAD) which captures up to 16 diagnosis fields for each hospitalization using ICD-9 for the years 1992/93 through 2000/01 and up to 25 diagnosis fields using ICD-10-CA thereafter.

1. Tu K, Jaakkimainen L, Butt D. Validation of Administrative Data Algorithms to determine Population Prevalence and Incidence of Alzheimer's Disease, Dementia, Multiple Sclerosis, Epilepsy and Parkinson's Disease. National Population Health Study of Neurological Conditions. Ottawa: Public Health Agency of Canada, 2013.

Dementia Incidence and Mortality

Exhibit 3 - Incident rate and mortality hazard for people with dementia				
	Incident rate		Mortality Hazard	
	Per person/year		versus people without demtia	
**Age**	Female	Male	Female	Male
**40-44**	0.000110	0.000226	8.26	19.1
**45-49**	0.000186	0.000207	6.92	10.5
**50-54**	0.000244	0.000278	5.92	6.67
**55-59**	0.000365	0.000448	6.76	6.23
**60-64**	0.000768	0.000909	6.27	5.00
**65-69**	0.001948	0.002359	4.61	3.92
**70-74**	0.005547	0.005921	3.40	3.30
**75-79**	0.013558	0.012819	2.74	2.53
**80-84**	0.026079	0.024987	2.46	2.09
**85-89**	0.048040	0.043578	2.38	1.37
**90-94**	0.048040	0.043578	1.47	0.90
**95+**	0.048040	0.043578	0.89	0.56

Exhibit 4 – Dementia incidence rate, annual incident rate, per person/year, males and females

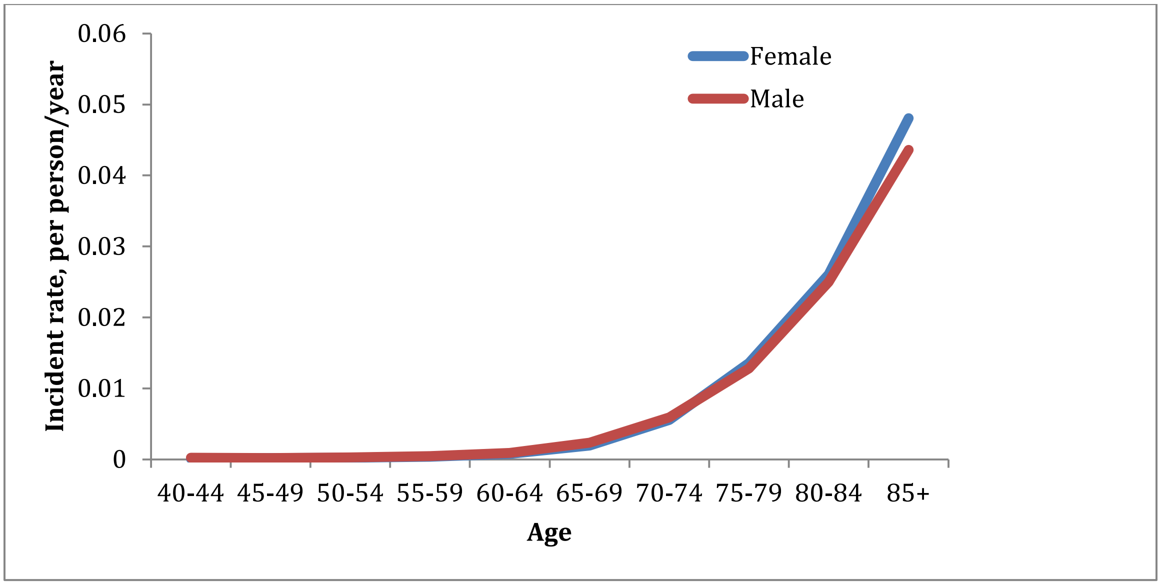

Exhibit 5 – Dementia mortality hazard, people with dementia versus people without dementia, males and females

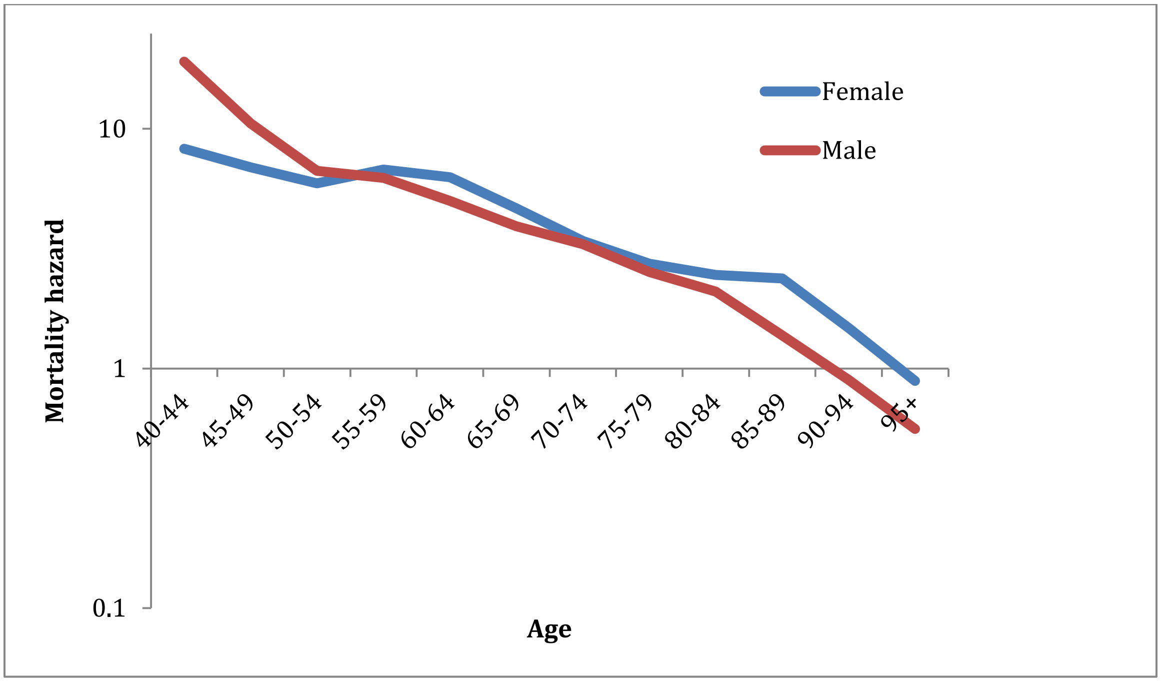

Health care costs

Costs were examined across seven broad categories: (i) physician services; (ii) prescription pharmaceuticals; (iii) hospitalizations; (iv) rehabilitation; (v) home care services; (vi) long-term care; and (vii) assistive devices.

Exhibit 3 - Health care costs: General methodology

POHEM-Neurological parameter: AverageCost	
**Population**	All individuals
**Sex**	Males, Females
**Ages**	5-year age groups
**Other**	8 groups: Each of the seven priority neurological conditions, and a cohort of individuals without priority neurological conditions<br><br>2 condition-status groups: (i) incident cases; (ii) prevalent cases<br><br>2 provinces: (i) British Columbia; (ii) Ontario<br><br>7 health sectors

POHEM-Neurological parameter: cost_prop_from_BC	
**Population**	All individuals
**Sex**	
**Ages**	
**Other**	A single cell into which the weight given to health care cost information when weighting costs from Ontario and British Columbia

Cost data were provided by both British Columbia and Ontario for the following health sectors: (i) physician services; (ii) prescription pharmaceuticals; (iii) hospitalization; and

(iv) rehabilitation. For the remaining health sectors, cost data were provided only for Ontario. Where cost data were available for both provinces, a weighted average of the two values was implemented in POHEM-dementia (Equation 1 for weighted mean, Equation 2 for weighted standard error).

Equation 1. Calculation of weighted average cost, weighting estimates from British Columbia and Ontario

$$weighted\ average\ cost = \frac{(cost\_prop\_from\_BC)(average\ cost_{BC}) + (1 - cost\_prop\_from\_BC)(average\ cost_{ON})}{2}$$

Equation 2. Calculation of weighted standard error of cost, weighting estimates from British Columbia and Ontario

$$weighted\ SE\ cost = \sqrt{(cost\_prop\_from\_BC)^2(SE\ cost_{BC})^2 + (1 - cost\_prop\_from\_BC)^2(SE\ cost_{ON})^2}$$

The weight used reflects the relative size of each province’s population. More precisely, the POHEM-Neurological parameter <cost_prop_from_BC> is used to define the weight applied to cost estimates obtained from British Columbia, the complement being the weight applied to estimates from Ontario. Based on census data, in the models delivered to PHAC (December 2013), <cost_prop_from_BC> has a value of 0.25.

Exhibit 4 - Population counts for Ontario and British Columbia, 2006 Census and 2011 Census

	2006		2011	
	n	%	n	%
**Ontario**	12,160,282	74.7	12,851,821	74.5
**British Columbia**	4,113,487	25.3	4,400,057	25.5
**Total**	16,273,769	100.0	17,251,878	100.0

Note. The above census data excludes population counts for one or more incompletely enumerated Indian reserves or Indian settlements.

Pharmaceutical costs are only covered for all Ontarians age 65 and older, whereas all British Columbians are eligible for pharmaceutical coverage. Therefore, estimates of prescription pharmaceutical costs for individuals under age 65 in the microsimulation model are based solely on estimates from British Columbia. After age 65, the microsimulation model uses a weighted estimate of pharmaceutical costs using data from both provinces.

Note that, in the above situations, the "usable" cost estimates from one province are copied into parameter cells for the other province, so that all parameter cells contain an estimate.

In POHEM-Dementia, the mean yearly per capita costs from Ontario and British Columbia are stored in the parameter <AverageCost>. In the model, synthetic individuals are assigned "NoNeuro" costs specific to their age and sex. Incident costs are assigned to

synthetic individuals for the first 365 days following diagnosis of a neurological condition. For individuals who have been diagnosed with a priority neurological condition for more than one year, prevalent costs are assigned specific to their age and sex. The exception to this strategy is the assignment of costs associated with cerebral palsy. For this condition, incident costs will be attributed to all synthetic individuals with cerebral palsy under age 20. Starting at age 20 and thereafter, only prevalent costs will be implemented in the microsimulation model.

Exhibit 5 - Out-of-pocket expenses for individuals with priority neurological conditions

	POHEM-Dementia parameter: NEW_PAT_OOP
**Population**	Individuals with Dementia
**Sex**	No stratification by sex
**Ages**	No stratification by age
**Other**	3 HUI3 categories: (i) [min, 0.34), (ii) [0.34, 0.76), (iii) [0.76, 1.00]

Some health care services are covered by provincial health insurance plans. Others are covered by private health insurance. However, there are certain costs that are borne by the individual: these are called out-of-pocket costs. Because these costs are not found on any administrative database, survey data will be used to assess out-of-pocket expenses. Data for out-of-pocket expenses were estimated from the 2011 *Survey on Living with Neurological Conditions in Canada*. Respondents or a proxy respondent were asked if they had incurred any expenses related to their condition for which they did not expect to

be reimbursed. Categories of expenses that were specifically addressed in the survey were:

- prescription and non-prescription (over-the-counter) medications
- assistive devices such as mobility aids, agility aids or specialized equipment
- rehabilitation therapy such as physical, occupational, speech or massage therapy
- home care services such as health care, homemaker, or other support services
